# Thalamocortical Spectral Transmission Relies on Balanced Input Strengths

**DOI:** 10.1007/s10548-021-00851-3

**Published:** 2021-06-04

**Authors:** Matteo Saponati, Jordi Garcia-Ojalvo, Enrico Cataldo, Alberto Mazzoni

**Affiliations:** 1 The Biorobotics Institute, Department of Excellence in Robotics and AI, Sant’Anna School of Advanced Studies, Viale Rinaldo Piaggio 34, 56025 Pontedera, IT Italy; 2 Dipartimento di Fisica “E. Fermi”, Largo Bruno Pontecorvo 3, 56127 Pisa, IT Italy; 3 Department of Experimental and Health Sciences, Universitat Pompeu Fabra, Barcelona Biomedical Research Park Dr. Aiguader 88, 08003 Barcelona, ES Spain

**Keywords:** Thalamocortical system, Information transmission, Neural networks, Parametrical analysis, Collective dynamics

## Abstract

The thalamus is a key element of sensory transmission in the brain, as it gates and selects sensory streams through a modulation of its internal activity. A preponderant role in these functions is played by its internal activity in the alpha range ([8–14] Hz), but the mechanism underlying this process is not completely understood. In particular, how do thalamocortical connections convey stimulus driven information selectively over the back-ground of thalamic internally generated activity? Here we investigate this issue with a spiking network model of feedforward connectivity between thalamus and primary sensory cortex reproducing the local field potential of both areas. We found that in a feedforward network, thalamic oscillations in the alpha range do not entrain cortical activity for two reasons: (i) alpha range oscillations are weaker in neurons projecting to the cortex, (ii) the gamma resonance dynamics of cortical networks hampers oscillations over the 10–20 Hz range thus weakening alpha range oscillations. This latter mechanism depends on the balance of the strength of thalamocortical connections toward excitatory and inhibitory neurons in the cortex. Our results highlight the relevance of corticothalamic feedback to sustain alpha range oscillations and pave the way toward an integrated understanding of the sensory streams traveling between the periphery and the cortex.

## Introduction

During the past decades several key features of sensory processing in the primary sensory cortex have been discovered, but much less is known about sensory information processing and transmission in the thalamus (Rikhye et al. 2018). The majority of sensory signals are conveyed by the thalamus to the cortex in the form of spiking patterns propagating along different pathways (Kandel et al. 2000). Thalamocortical relay neurons in the thalamus receive sensory inputs and in turn project them to particular areas of the cortex through thalamocortical synapses. For decades, the thalamus has been described as a relay station where little computation takes place. However, more recently, experimental and theoretical findings have shown a prominent role of the thalamus in both pre-processing of sensory stimuli (Roth et al. 2016) and modulation of cortical activity even in absence of external stimulation (Steriade et al. 1993; Constantinople and Bruno 2013; Reinhold et al. 2015). Here we investigate the interplay of these two functions of the thalamus, using a novel spiking network model of the two areas.

Several brain structures exhibit oscillatory activity at different timescales related to various cognitive functions such as memory consolidation (Diekelmann and Born 2010), attention (Gray and Singer 1989; Lakatos et al. 2008) and information transmission (Klimesch 2012). State-of-the-art techniques in systems neuroscience provides a detailed description of anatomical and electrophisiological structures of thalamic and cortical systems. However, a rigorous analysis of the role of thalamic and cortical oscillations in information transmission is missing. During sleep or anesthesia, slow-waves [0.1–1 Hz or 1–4 Hz] are present in both thalamic and cortical activities and seem to coherently organize the dynamics of both networks (Sherman 2007). On the other hand, fast rhythms in different frequency bands characterize the activity of the thalamocortical system during states of awakeness or REM sleep. The thalamus also shows rhythmic activities in the [8–12 Hz] range. These oscillations are present during sleep in an intermittent way (Steriade et al. 1993) and they are called spindle oscillations. In the awake state different biophysical mechanisms originate more continuous oscillations in a very similar range, called alpha rhythms (Neuronal Mechanisms 2011). Both these oscillations tend to co-occur in the cortex, but it is unclear to which extent this co-occurrence is due to a cortical entrainment to external input and/or modulation of cortical circuits with similar resonances, and/or generated through corticothalamic feedback (Da Silva et al. 1973). Shedding light on these mechanisms would also clarify why these frequencies are sometimes dissociated between cortex and thalamus in the awake state (e.g., Bastos et al. (2014)). Here we focus on investigating the first mechanism, i.e, on understanding to which extent frequencies in the alpha range [8–12] Hz are transmitted from thalamus to cortex in a feedforward way. Indeed, the joint thalamocortical system includes neural rhythms at different frequencies, which will have differential impact on the frequency bands that are known to carry different information in the cortex (Mazzoni et al. 2008).

**Fig. 1:**
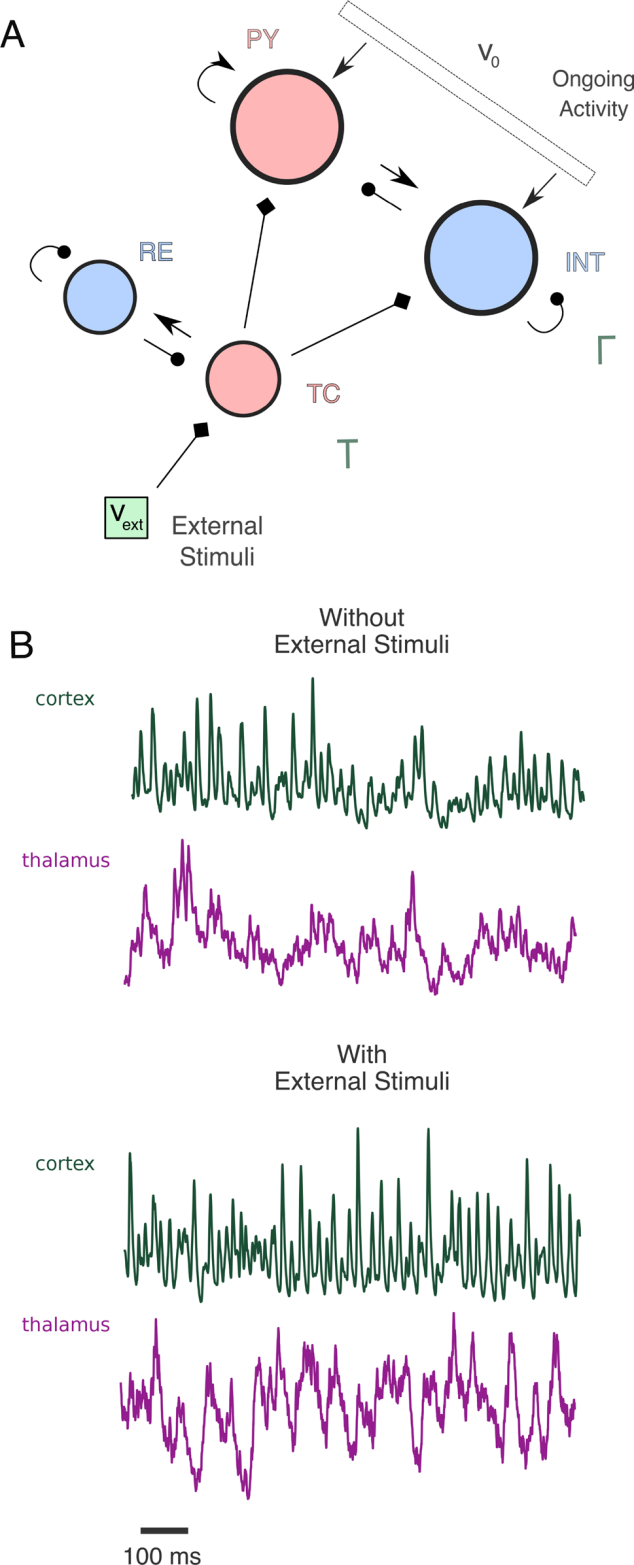
Network design and dynamics. **A** Representation of the network structure. The thalamocortical model is composed by a thalamic network $\text {T}$ and a cortical network $\Gamma$. Both networks are divided into an excitatory and an inhibitory population, in red and blue respectively. Arrows with triangle-shaped and circle-shaped heads represent excitatory and inhibitory connections, respectively. Arrows with diamond-shaped heads represent excitatory connections between different areas (External Stimuli to TC population, TC population to cortical populations). The cortical network receives background excitatory stimulation simulating ongoing activity of afferent cortical areas. **B** Temporally aligned cortical $\text {LFP}_{\Gamma }$ (green) and thalamic $\text {LFP}_{\text {T}}$ (purple) from a numerical simulation in the absence ($\nu _{\text {ext}} =$ 0 spk/ms) or presence ($\nu _{\text {ext}} =$ 0.5 spk/ms) of external stimuli (see “Methods”)

Information throughout the brain is mainly transferred by excitatory populations through synaptic connections between different areas. In the thalamus, links from sub-cortical areas to the cortex are driven by AMPAergic thalamocortical relay (TC) neurons. These excitatory neurons are surrounded by a shell of GABAergic reticular neurons (RE) which do not receive sensory input directly (see Fig. 1A) and are supposed to modulate the information flow (Steriade 2005). They receive afferents from TC neurons and send inhibitory inputs back to them, creating a closed-loop between thalamic population. Therefore, the activity of TC neurons does not convey a faithful reproduction of sensory information, but rather a pre-processed version of them in function of internal states of the thalamus. Intrinsic features of thalamocortical feedforward connectivity are indeed crucial in shaping this information transmission. However, a complete understanding of anatomical and functional connectivity from TC neurons to the cortex is missing.

In this scenario, we aim to shed new light on thalamic and cortical spindle oscillations basing our investigations on a recently developed integrate-and-fire network model (Saponati et al. 2019) able to reproduce network oscillations on a wide range of timescales.

## Methods

Here we summarize the main features of the model: the network is an extension of a thalamic network model introduced in Barardi et al. (2016) connected to a previously developed cortical network model Mazzoni et al. (2008, 2011).

### Neural and Network Models

The thalamocortical network model consists of two structures, namely a thalamic network $\text {T}$ and a cortical network $\Gamma$, see Fig. 1A for a graphical representation of network structure. Both networks are composed by an excitatory and an inhibitory population. The thalamic network $\text {T}$ is composed by 250 *thalamocortical relay* (TC) neurons with AMPA-like synapses and 250 *reticular* (RE) neurons with GABA-like synapses. The cortical network $\Gamma$ is composed by 4000 *pyramidal* (PY) neurons with AMPA-like synapses and 1000 *interneurons* (INT) with GABA-like synapses. Thalamic and cortical structures are characterized by random and sparse connectivity schemes with different coupling probabilities. Any directed pair of cortical neurons are connected with a probability *p* = 0.02 independently from the neuron type, while thalamic RE neurons connect with TC neurons with a probability *p* = 0.04 and TC neurons connect to RE neurons with *p* = 0.01. Moreover, RE network structure shows recurrent connections with probability *p* = 0.04 on which we add a degree of clustering by starting from a ring network and then randomly rewiring with probability 0.25. This is necessary for the onset of sustained thalamic oscillations (Barardi et al. 2016). The model includes thalamocortical afferents by considering synaptic connections from TC neurons to cortical excitatory and inhibitory populations. Thalamocortical connectivity is random and sparse with a connection probability *p* = 0.07.

**Table 1:** Single-neuron parameter set for every population considered in the model

	Units	PY	INT	TC	RE
$\tau _m$	ms	20	10	25	25
$\tau _{r}$	ms	2	2	2.5	2.5
$E_l$	mV	− 60	− 60	− 70	− 70
$\Delta$	mV	2.5	2.5	2.5	2.5
$\Theta _{th}$	mV	− 52	− 52	− 50	− 50
$$v_{reset} $$	mV	− 59	− 59	− 60	− 60
$g_l$	nS	25	20	50	50
*a*	nS	1	1	0.2	0.4
*b*	nA	0.6	0	0	0.02
$\tau _w$	ms	600	600	600	600

We consider these values for all numerical simulations of the manuscript, unless otherwise stated. The parameter values are adapted from Saponati et al. (2019)

**Table 2:** Synaptic parameter sets for every coupling between population considered

	Units	PY,PY	INT,PY	PY,INT	INT,INT	RE,TC	TC,RE	RE,RE	PY,TC	INT,TC
*p*		0.2	0.2	0.2	0.2	0.01	0.04	0.04*	0.07	0.07
$\bar{g}$	nS	0.178	0.233	2.10	2.70	0.2	0.3	0.3	3.28	4.44
$\tau _{\uparrow }$	ms	0.4	0.2	0.25	0.25	0.4	0.4	0.4	0.4	0.2
$\tau _{\downarrow }$	ms	2	1	5	5	5	10	10	2	1
*E*	mV	0	0	− 80	− 80	0	− 80	− 80	0	0
$\tau$	ms	1	1	1	1	1	1	1	1 $\div$ 2	1 $\div$ 2

Recurrent connections between RE neurons are defined by random and sparse connectivity scheme superimposed on a small-network arrangement (Barardi et al. 2016). We will use this parameter set throughout this manuscript, unless otherwise stated. The parameter values are adapted from Saponati et al. (2019)

Neuronal dynamics is simulated with the Adaptive Exponential Integrate-and-Fire model (Brette and Gerstner 2005)

1 $$\begin{aligned} \tau _m\frac{dv_{i,\alpha }}{dt}&= -(v_{i,\alpha } - E_l) + \Delta \exp \Big (\frac{v_{i,\alpha }-v_{th}}{\Delta }\Big ) \nonumber \\&\quad - \frac{1}{g_l}w_{i,\alpha } + \frac{1}{g_l}\sum _{\beta ,j}C_{ij}^{\alpha ,\beta }I_{ij}^{\beta }\end{aligned}$$

2 $$\begin{aligned} \tau _w \frac{dw_{i,\alpha }}{dt}&= a(v_{i,\alpha } - E_l) - w_{i,\alpha } \end{aligned}$$

where the sub-threshold dynamics of *i*-th neurons of a certain population $\alpha$ (excitatory or inhibitory population of cortical or thalamic network) is described by two coupled state variables $\big (v_{i,\alpha }(t),w_{i,\alpha }(t)\big )$, the membrane potential and the adaptation variable, respectively, endowed with discrete reset dynamics. In particular, $\tau _m$ is the membrane time constant, $E_l$ is the reversal membrane potential and $g_l$ is the membrane leak conductance. Table 1 contains all the values considered in this study for the parameters of the Adaptive Exponential Integrate-and-Fire model. The activity of the *i*-th neuron in population $\alpha$ is the collection of emitted spikes $\{t_{i,\alpha }\}$ over time $\rho _{i,\alpha }(t) = \sum _{\{t_{i,\alpha }\}} \delta (t - t_{i,\alpha })$. The synaptic current $I_{ij}^{\alpha ,\beta }$ represents the input to the *i*-th neuron of population $\alpha$ given by the activity of *j*-th pre-synaptic neuron belonging to the population $\beta$. This current is described with a double-exponential conductance-based model

3 $$\begin{aligned} \begin{aligned} I^{\alpha ,\beta }_{ij}(t)&= \bar{g}_{\alpha ,\beta }\big (v_{i,\alpha } - E_{\beta }\big )\sum _{t_k \in \rho _{j,\beta }}\bigg [\exp \bigg (-\frac{t - t_k - \tau }{\tau _{\uparrow }}\bigg )-\\&\quad - \exp \bigg (-\frac{t - t_k - \tau }{\tau _{\downarrow }}\bigg )\bigg ] \end{aligned} \end{aligned}$$

where $\bar{g}_{\alpha ,\beta }$ is the maximal conductance, $E_{\beta }$ is the synaptic reversal potential (which value is determined only by the population $\beta$ of the pre-synaptic neuron), $\tau$ is the propagation delay and $\tau _{\uparrow }$ and $\tau _{\downarrow }$ are the rise and decay time constant, respectively. Every neuron receives a total synaptic current which is the linear sum of such contributions. A coupling matrix $C^{\alpha \beta }$ defines the connections from population $\beta$ to population $\alpha$. All parameter sets have been chosen accordingly with literature and recent experimental findings (Sedigh-Sarvestani et al. 2017), for further details and parameter values used refer to Saponati et al. (2019). Table 2 contains the parameter values of the synaptic model considered in this study.

Every TC neuron receives an external excitatory input mimicking sensory signals. Every cortical neuron receives an external excitatory input mimicking ongoing activity from afferent cortical areas together with the thalamocortical input. Both external inputs are simulated as Poisson spike trains with different rate parameters. In particular, stimulus unrelated activities from afferent cortical areas are given by different time-varying rates $\nu _0(t)$ following an Ornstein–Uhlenbeck process

4 $$\begin{aligned} \tau _n \frac{d\nu _0(t)}{dt} = - \big (\nu _0(t) - \bar{\nu }_0\big ) + \sqrt{\frac{2}{\tau }_n}\sigma _n\eta (t) \end{aligned}$$

where $\eta (t)$ is a Gaussian white noise, $\tau _n$ is the characteristic time of the stochastic process and $\bar{\nu }_0$ = 0.75 spk/ms and $\sigma _n$ = 0.5 spk/ms are the mean and standard deviation, respectively. The parameter values of the stochastic input rate have been chosen in order to match with experimental observations in V1 during external stimulation (Mazzoni et al. 2010). Inputs coming from the sensory system are simulated as homogeneous Poisson spike trains with different constant rate values $\nu _{\text {ext}}$ ranging from 0 to 1 spk/ms. We consider two different regimes: one without sensory inputs ($\nu _{\text {ext}}$ = 0 spk/ms) and one with TC neurons receiving external inputs of different rates ($\nu _{\text {ext}}$ > 0 spk/ms). All synaptic currents from external sources are modeled with a double-exponential conductance-based model as in (3). We used the same parameter values of the intra-network excitatory synapses (see Table 2) for the external excitatory inputs to the respective thalamic and cortical network.

### Computation of Simulated LFP and Network Firing Rate

We define a field quantity related to experimentally recorded mesoscopic LFP signals (Buzsáki et al. 2012; Einevoll et al. 2013; Pesaran et al. 2018). Following (Saponati et al. 2019), we compute cortical $\text {LFP}_{\Gamma }(t)$ and thalamic $\text {LFP}_{\text {T}}(t)$ as linear combinations of all synaptic intra-network currents. In particular, we sum the absolute values of all synaptic currents to pyramidal (PY) neurons for cortical LFP, and the absolute values of all synaptic currents between TC and RE neurons for thalamic LFP

5 $$\begin{aligned} \text {LFP}_{\Gamma }(t)= & {} \sum _{i=1}^{N_{PY}}\sum _{j=1}^{N_{PY}}\big |I_{ij}(t)\big |^{PY,PY} + \sum _{i=1}^{N_{PY}}\sum _{j=1}^{N_{INT}}\big |I_{ij}(t)\big |^{PY,INT} \end{aligned}$$

6 $$\begin{aligned} \text {LFP}_{\text {T}}(t)= & {} \sum _{i=1}^{N_{RE}}\sum _{j=1}^{N_{TC}}\big |I_{ij}(t)\big |^{RE,TC} + \sum _{i=1}^{N_{TC}}\sum _{j=1}^{N_{RE}}\big |I_{ij}(t)\big |^{TC,RE} +\nonumber \\&\quad + \sum _{i=1}^{N_{RE}}\sum _{j=1}^{N_{RE}}\big |I_{ij}(t)\big |^{RE,RE} \end{aligned}$$

This simple method of computing LFPs from spiking network models is robust and efficient under the assumption of homogeneous extracellular medium, as shown in Mazzoni et al. (2015). We compute the firing rate of each neural network as the mean of firing rates of all neurons from both excitatory and inhibitory populations

7 $$\begin{aligned} \text {FR}(t) = \sum _{\alpha }\frac{1}{N_{\alpha }}\sum _{i = 1}^{N_{\alpha }} \text {FR}_{i,\alpha }(t) = \sum _{\alpha }\frac{1}{N_{\alpha }}\sum _{i = 1}^{N_{\alpha }} \int _{t}^{t + dt}\rho _{i,\alpha }dt \end{aligned}$$

where $\alpha$ runs over both excitatory and inhibitory populations. We express thalamic and cortical firing rates in number of spikes per millisecond (spk/ms). We also performed spectral analysis of the firing rate to focus on the output of neuron sets, as the LFP primarily reflects the synaptic input in our model (Mazzoni et al. 2015).

### Spectral Analysis

LFP power spectral densities are estimated from a dataset of 40 different simulations of length *T* = 10 s, each generated with different noise realisations. We compute the power spectral density (PSD) with a FFT using Welch method (*pwelch* function in MATLAB). To that end, the LFP signal is split up into 8 sub-windows with $50\%$ overlap. The overlapping segments are windowed with an Hamming window. The periodogram is calculated by computing the discrete Fourier Transform, and then calculating the square magnitude of the result. The modified periodograms are then averaged to obtain the power spectral density estimate. We compute the magnitude-squared coherence between the thalamic and cortical LFP signals estimated from the same dataset again using the Welch’s method (*mscohere* function in MATLAB)

8 $$\begin{aligned} C_{\Gamma ,\text {T}}(f) = \langle \frac{\big |\text {PSD}_{\Gamma ,\text {T}}(f)\big |^2}{\text {PSD}_{\Gamma ,\Gamma }(f)\text {PSD}_{\text {T},\text {T}}(f)}\rangle \end{aligned}$$

where $\text {PSD}_{\Gamma ,\text {T}}(f)$ is the estimated cross-spectrum, $\text {PSD}_{\Gamma ,\Gamma }(f)$ and $\text {PSD}_{\text {T},\text {T}}(f)$ are respectively the estimated cortical and thalamic PSDs and $\langle \,\, \rangle$ is the average over the dataset. We also analyze the distribution of phase lags in frequency-bands of interest, to investigate possible synchronizations of the two networks activities. The phase relation between Fourier-transformed signals $\text {LFP}^*_{\text {T}}(f)$ and $\text {LFP}^*_{\Gamma }(f)$ at a given frequency *f* is quantified as the angle of the estimated complex-value cross-spectrum, similarly to (Womelsdorf et al. 2007). We compute phase-lags $\Delta \phi _i(f)$ for every *i*-th simulation in the dataset, and average over frequency-bands of interest. The frequency bands of interest are: $\delta$ [1–4] Hz, $\alpha$ [8–12] Hz, $\beta$ [13–30] Hz, $\gamma$ [30–80 Hz]. Please note that the $\alpha$ band includes in its range both proper $\alpha$ oscillations, present in the thalamus in the awake state (Neuronal Mechanisms 2011) and the intermittent spindle oscillations, present in the thalamus in sleep state (Contreras et al. 1997), and it is therefore very relevant to assess the properties of spectral thalamocortical transmission of this band. We interpret the estimated phase relations as directional data and, for every frequency-band considered, we compute the circular variance (Mardia and Jupp 2009)

9 $$\begin{aligned} r = \bigg |\frac{1}{N}\sum _{i=1}^N \mathbf {r}_i\bigg | = \bigg |\frac{1}{N}\sum _{i=1}^N \begin{bmatrix}\cos (\Delta \phi _i)\\ \sin (\Delta \phi _i)\end{bmatrix}\bigg | \end{aligned}$$

that is, the mean resultant vector modulus of the set. We test the presence of a preferred phase-lag in a certain frequency band with a Rayleigh test between a uniform distribution hypothesis $H_0$ and a non-uniform distribution hypothesis $H_1$, considering the approximated p-value (Zar 2013)

10 $$\begin{aligned} p = \exp \bigg ({\sqrt{1 + 4N+ 4(N^2 - r_n^2)} - (1 + 2N)}\bigg ) \end{aligned}$$

where *N* is the number of sample in dataset and $r_n = rN$. With such a method we quantify if phase-lag values are given by random fluctuations, or whether they belong to a certain non-uniform distribution (Berens 2009). We compute filtered Local Field Potential signals in the $\delta$ and $\alpha$ ranges by means of a digital filter (*designfilt* function in MATLAB). In particular, we consider the cut-off frequencies to be 1 Hz and 4 Hz for the $\delta$-range, 8 Hz and 12 Hz for the $\alpha$-range. We use the MATLAB function *filtfilt*, which processes the input data in both the forward and reverse directions to eliminate the phase lag.

### Numerical Methods

All scripts are written in MATLAB. The dynamical equations of the system are solved numerically with a second-order Runge–Kutta method with *mid-point* scheme and time step *h* = 0.05 ms. Some scripts have been implemented with the DynaSim Toolbox (Sherfey et al. 2018).

## Results

We investigated in silico the properties of thalamocortical information transmission through our local network model of the thalamus receiving external stimuli (Barardi et al. 2016) and projecting to the primary cortex, which receives also stimulus-unrelated excitatory inputs (Mazzoni et al. 2011) (Fig. 1A). We computed the cortical $\text {LFP}_{\Gamma }(t)$ and the thalamic $\text {LFP}_{\text {T}}(t)$ (Mazzoni et al. 2015) (Fig. 1B, see “Methods” for details) as main outputs of the system.

### Frequency-Dependent Transmission in Thalamocortical Connections

**Fig. 2:**
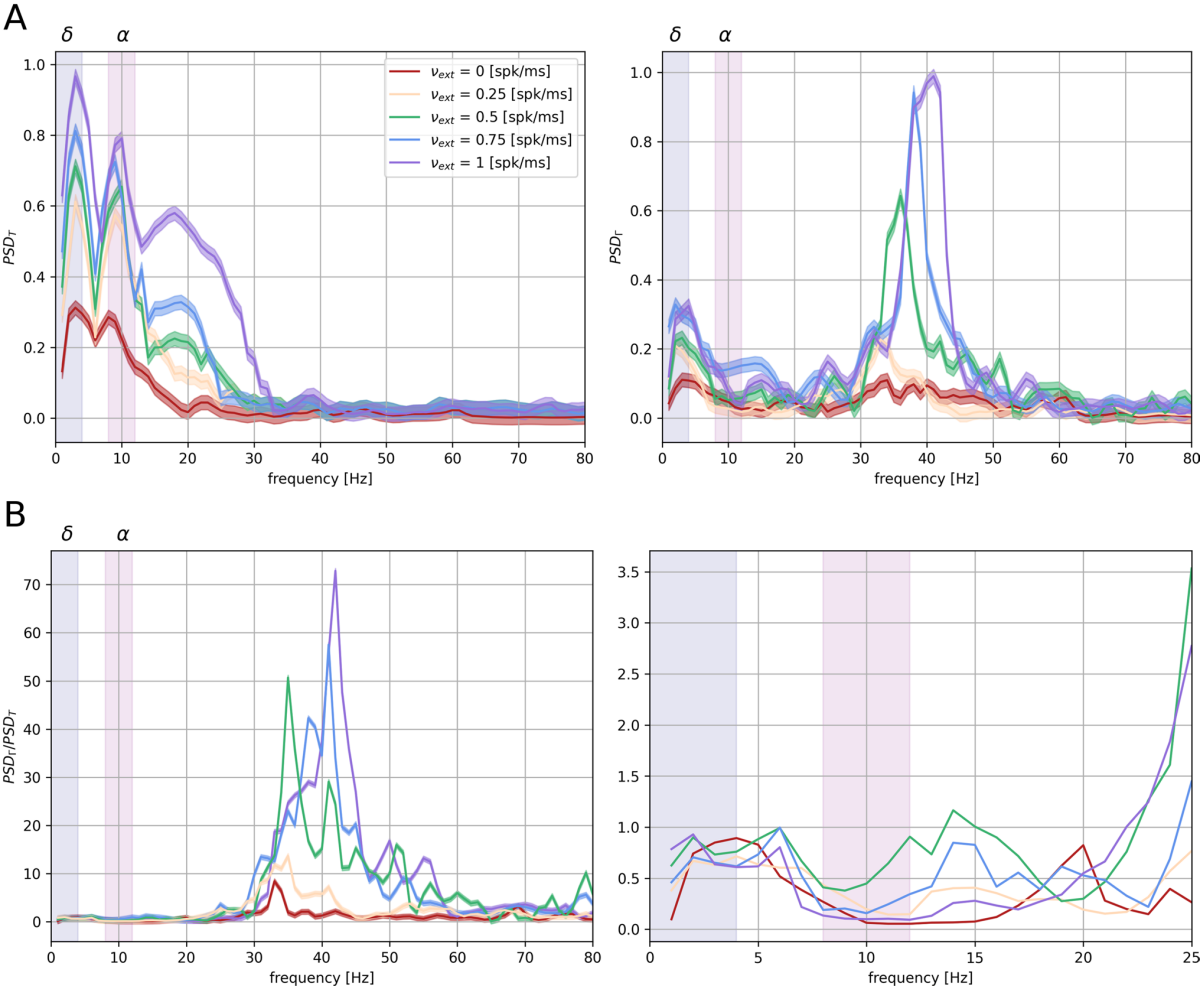
Modulation of thalamocortical LFP spectrum by external input. **A** Estimated power spectral densities $\text {PSD}_{\text {T}}$ and $\text {PSD}_{\Gamma }$ of thalamic and cortical LFP for different external stimulation (the different input intensities are indicated in the legend). The results are averaged over 40 simulations with different noise realisations and normalized. Each synaptic input is simulated as an homogeneous Poisson spike-train with rate $\nu _{\text {ext}}$ (see “Methods”). The blue and purple stripes represent $\delta$ [1–4 Hz] and $\alpha$ [8–12 Hz] frequency range, respectively. **B** Ratio between cortical and thalamic PSD as a function of input rate. Right plot shows a zoom in the 0–25 Hz range of the same ratio between cortical and thalamic PSDs

**Fig. 3:**
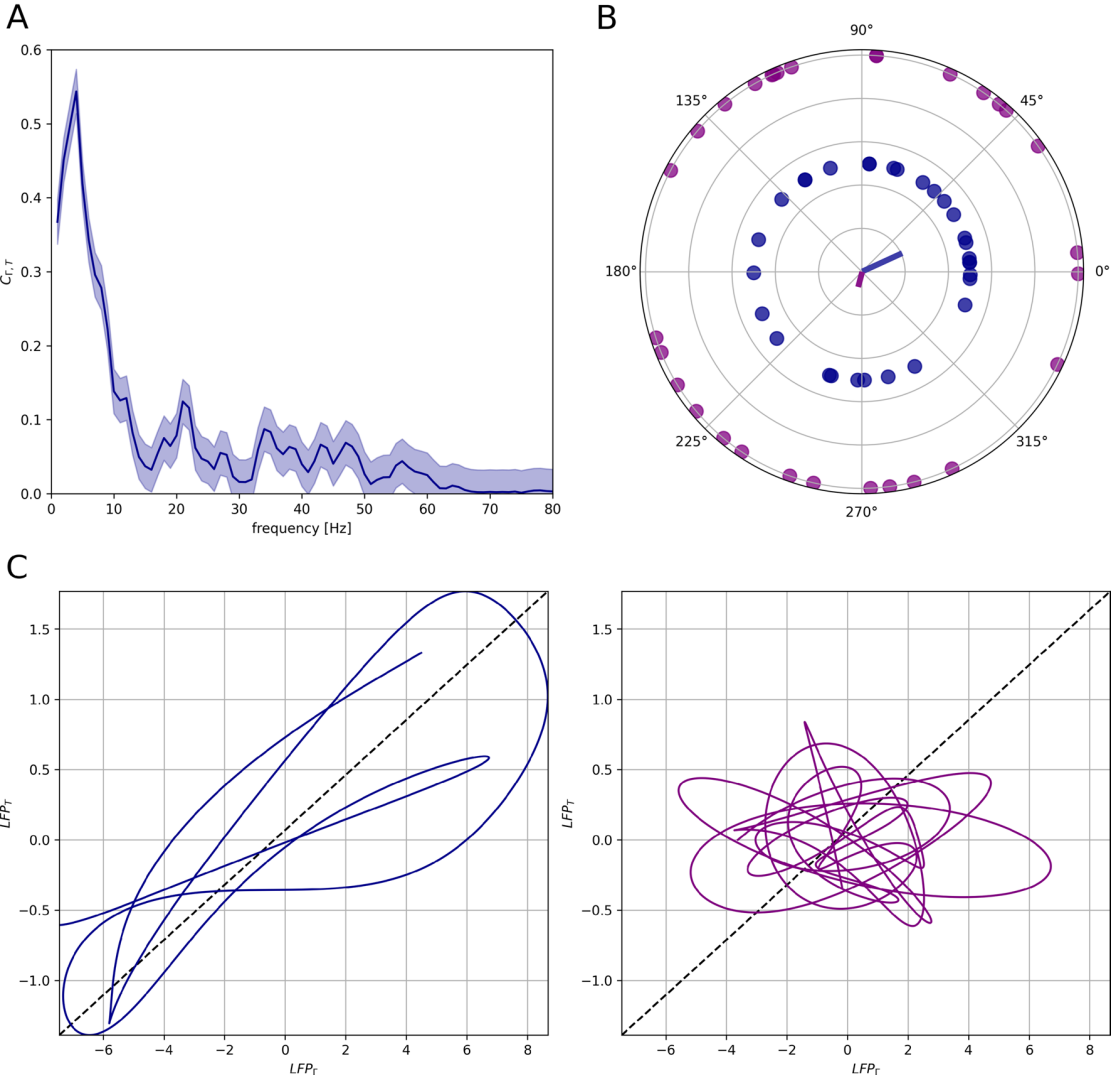
Phase relation between the cortical and thalamic LFPs. **A** Magnitude squared coherence (see “Methods”) between thalamic and cortical networks during external stimulation $\nu _{ext}$ = 0.5 spk/ms. **B** Circular scatterplot of phase-lags between cortical and thalamic LFP signals in the $\delta$ (1–4 Hz, blue) frequency band and in the $\alpha$ (8–12 Hz, purple) frequency band. Circles are datasets of 30 different simulations during external stimulation $\nu _{ext}$ = 0.5 spk/ms. Lines in the center of the figures are the resultant vector lengths estimated from the dataset. C) Signal trajectory in the ($\text {LFP}_{\Gamma }$, $\text {LFP}_{\text {T}}$) space for two frequency bands of interest. The blue and purple trajectories represent filtered LFP in the $\delta$ and $\alpha$ ranges, respectively. Both filtered signals are obtained from the same raw LFP signal in a time-window of length *T* = 1 s during external stimulation $\nu _{ext}$ = 0.5 spk/ms

To capture the mechanisms underlying the relationship between spectral content in the thalamus (both input driven and internally generated) and in the cortex, we first compared the thalamic $\text {PSD}_{\text {T}}$ and the associated cortical $\text {PSD}_{\Gamma }$ from the respective LFPs both without external stimuli and during external stimulation, see “Methods” and (Barardi et al. 2016). In the former case both thalamic and cortical networks are associated to prominent $\delta$ [1–4 Hz] fluctuations, but display several secondary peaks, in the $\alpha$-band [8–12 Hz] and $\gamma$-band [30–80 Hz] respectively (Fig. 2A). When introducing external inputs, the gamma peak becomes more pronounced (Mazzoni et al. 2008) in the cortex, while a secondary peak in the beta range [13–30] Hz appears in the thalamus (Fig. 2B). The corticothalamic ratio, that is, the ratio of the cortical and the thalamic spectra across the stimulation range (see “Methods”), shows that both the $\alpha$-peak and the $\beta$-peak are weaker in the cortex (Fig. 2B), consistently with experimental observations (Bastos et al. 2014). This suggests that these bands are specifically suppressed by thalamocortical transmission. We observe that this suppression is poorly modulated by external inputs (Fig. 2B). Likewise, external inputs do not affect thalamocortical coupling in slower $\delta$-rhythms, which seem to be an intrinsic feature of the system, unrelated to stimuli. External inputs modulation is visible, instead, in the cortical $\gamma$-band activity, which is increasing proportionally to the input rate (Fig. 2B) (Henrie and Shapley 2005; Mazzoni et al. 2008, 2011). This modulation happens through enhancement of thalamic activity as shown in Fig. 2B. The local origin of gamma oscillations in cortical networks is a well known phenomenon (Sohal 2016). Lower cortical frequencies are instead supposed to be phase-locked to thalamic stimuli (Lakatos et al. 2005; Mazzoni et al. 2008; Lakatos et al. 2008; Mazzoni et al. 2011), as is the case in this model (see Fig. 3). However, the lack of entrainment of the cortical network to the thalamic inputs between the $\delta$ and the $\gamma$ bands is a less obvious phenomenon with important consequences for information transmission. In fact, while $\delta$-fluctuations are an important component of thalamic activity and they can convey information about the external world during stimulus-driven regimes (Schroeder and Lakatos 2009; Mazzoni et al. 2011) or about the internal brain state during sleep or anesthesia (Steriade et al. 1993; Lewis et al. 2015), $\alpha$/spindle-oscillations are locally originated in the thalamus (Steriade et al. 1985; Bazhenov et al. 2000) and hence do not carry information about the external world. Actually, thalamic spindle-rhythms interpose to information transmission and therefore contribute to the gating role of the thalamus (Sherman 2001). Therefore, our results shown in Fig. 2 suggest that there are mechanisms in the thalamocortical transmission that implicitly select the informative frequency bands. In the following we will investigate possible mechanisms underlying this selection.

### Phase Relationship Between Thalamocortical LFP

We asked then whether thalamic inputs regulate cortical activity by influencing its spectral content. We performed a band-wise coherence analysis (see “Methods”), focusing on the frequency bands of interest. We found that $\delta$-oscillations are coherent between the thalamic and cortical LFPs (Fig. 3A), in agreement with experimental analysis (Mazzoni et al. 2008), while the system does not show significant coherence in any other frequency bands. Moreover, phase lags in the $\delta$ band from different simulations tend to cluster around a mean-value, showing that the $\delta$ oscillations in the cortex are locked to those in the thalamus (Fig. 3B). We quantified this observation by computing the resultant vector lengths (see “Methods”): $R_{\delta }$ = 0.27 and $R_{\alpha }$ = 0.11 (vectors in Fig. 3B). We tested the uniformity of the sample distributions with a Rayleigh test (*p* = 0.05, see “Methods”): $p_{\delta }$ < 0.01 and $p_{\alpha }$ = 0.78. We then investigated the thalamic and cortical LFP signals filtered in the $\delta$ and $\alpha$ bands to qualitatively illustrate the different relationship between thalamus and cortex in the two bands (see “Methods”). The thalamic and cortical LFPs show a certain level of phase coupling in the $\delta$ range while they behave independently in the $\alpha$ range (Fig. 3C). These results show a strong $\delta$ coupling between the thalamic and cortical networks, while the spectral content in any other frequency band is not coherent within the thalamocortical system. In particular, intermediate $\alpha$ and $\beta$ rhythms in the thalamus do not entrain the corresponding bands in the cortex. We observe then that in the local patch of the cortex that we are simulating, alpha range oscillations are weaker than in the thalamus and do not have any phase relationship with the oscillations in the same range present in the thalamus. This strongly suggests that cortical input to reticular nucleus is necessary to ensure coherence in the alpha frequency range (Bollimunta et al. 2011). As before, this suggests that entrainment in $\delta$ band and filtering of $\alpha$ rhythms is a prominent characteristic of the thalamocortical system. Such role of slow thalamic rhythms in modulating cortical activity is a recurrent experimental finding (Lewis et al. 2015; Crunelli et al. 2018).

**Fig. 4:**
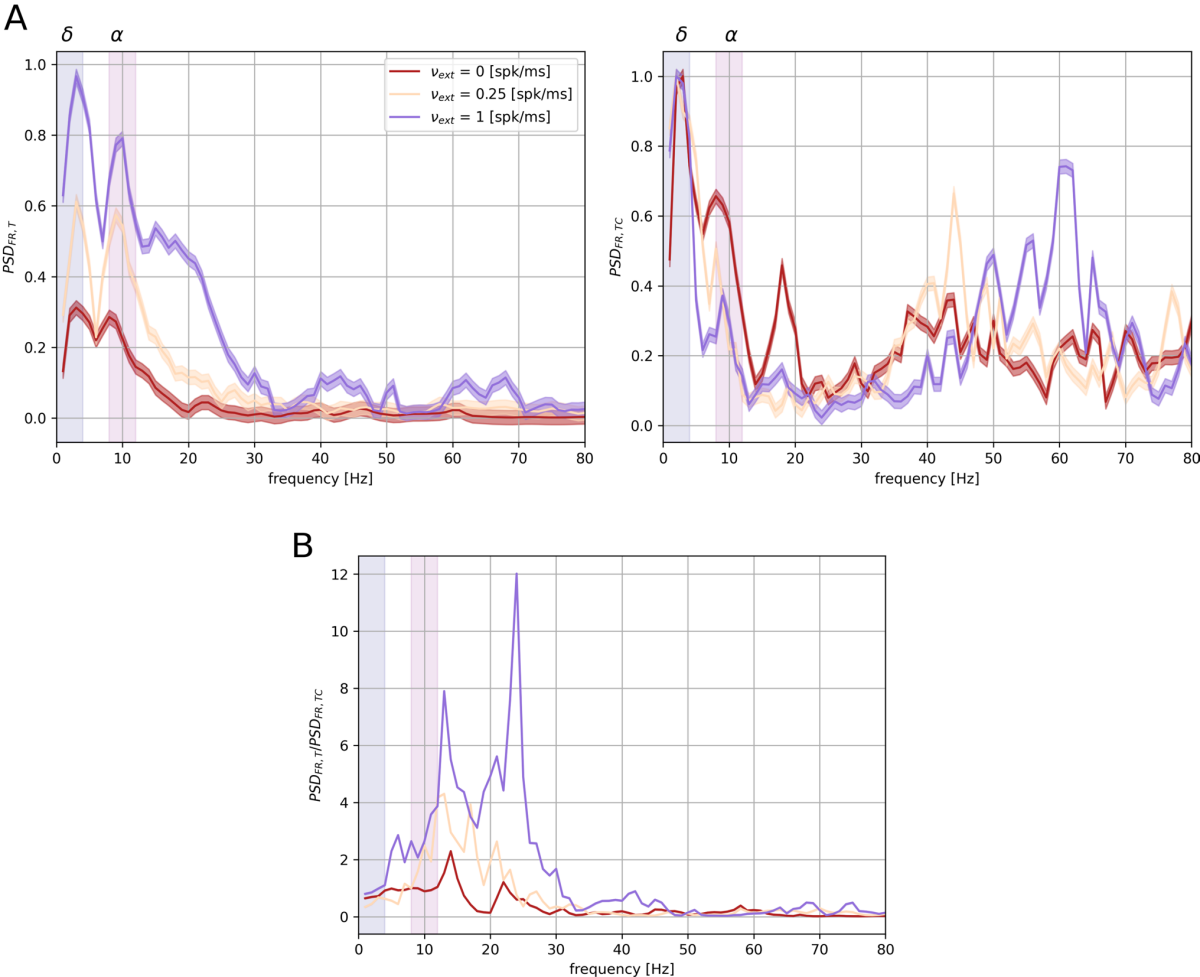
Spectral analysis of thalamic LFP and TC firing rate. Thalamic spectral features in function of different external stimulations (see legend). Blue and purple stripes represent $\delta$ [1–4 Hz] and $\alpha$ [8–12 Hz] frequency range, respectively. **A** Estimated power spectral densities $\text {PSD}_{\text {T}}$ and $\text {PSD}_{\text {FR},tc}$ of thalamic LFP (left) and TC firing rate (right). Results are averaged over 40 simulations with different noise realisations and normalized. **B** Ratio between thalamic LFP and TC firing rate PSDs

### Alpha Range Oscillations in TC Neurons

The different behaviors of the frequency bands in thalamocortical transmission necessarily depends on mechanisms at the thalamocortical projection level or inside cortical network dynamics. We focused in understanding these mechanisms by firstly characterizing alpha range ([8–12] Hz) oscillations in the subset of thalamic neurons projecting to the cortex, rather than in the overall thalamus dynamics. The LFP of a given network is determined by synaptic inputs (Buzsáki et al. 2012; Einevoll et al. 2013) and is simulated in our model accordingly (Mazzoni et al. 2015). Consequently, thalamic LFP is mainly composed of synaptic currents of RE inputs into TC neurons and recurrent RE-RE connections, because of their higher connection probabilities with respect to TC inputs (see “Methods”). However, thalamic inputs to the cortex are given only by the output of TC neurons. We compared the spectral features of thalamic $\text {LFP}_{\text {T}}$ and TC neurons firing at a rate $\text {FR}_{tc}$ (Fig. 4A, see “Methods”). Specifically, we computed the power spectral density of the thalamic LFP and the TC population firing rate, observing that the $\alpha$ component is much less pronounced in the latter than in the overall LFP. This suggests that part of the discrepancy between thalamic and cortical PSD in the $\alpha$ range (Bastos et al. 2014) is that only a weak component of thalamic spindle oscillations is transmitted to the cortex, as these oscillations are stronger in the RE than in the TC population (Barardi et al. 2016). Still, even considering the actual input to the cortex, the $\delta$ band is transmitted in a much more faithful way than the $\alpha$ oscillations (Fig. 4B). The ratio between the activities of the overall thalamic network and the TC population only shows increasing discrepancy in the $\alpha$ range, and particularly in the $\beta$ range, for increasing input rate. These discrepancies in the spectral content of network activities rely then on the thalamic connection scheme. The cortical spectrum, in the low frequency range, is indeed much more similar to the cortical input spectrum than to the overall thalamic LFP (Fig. 2A). The thalamus modulates cortical activity in the low $\delta$ rhythms in the absence and in the presence of external sensory stimulation, while processing the sensory input with internal spindle oscillations driven by RE inhibition, in agreement with experiments (Lewis et al. 2015).

**Fig. 5:**
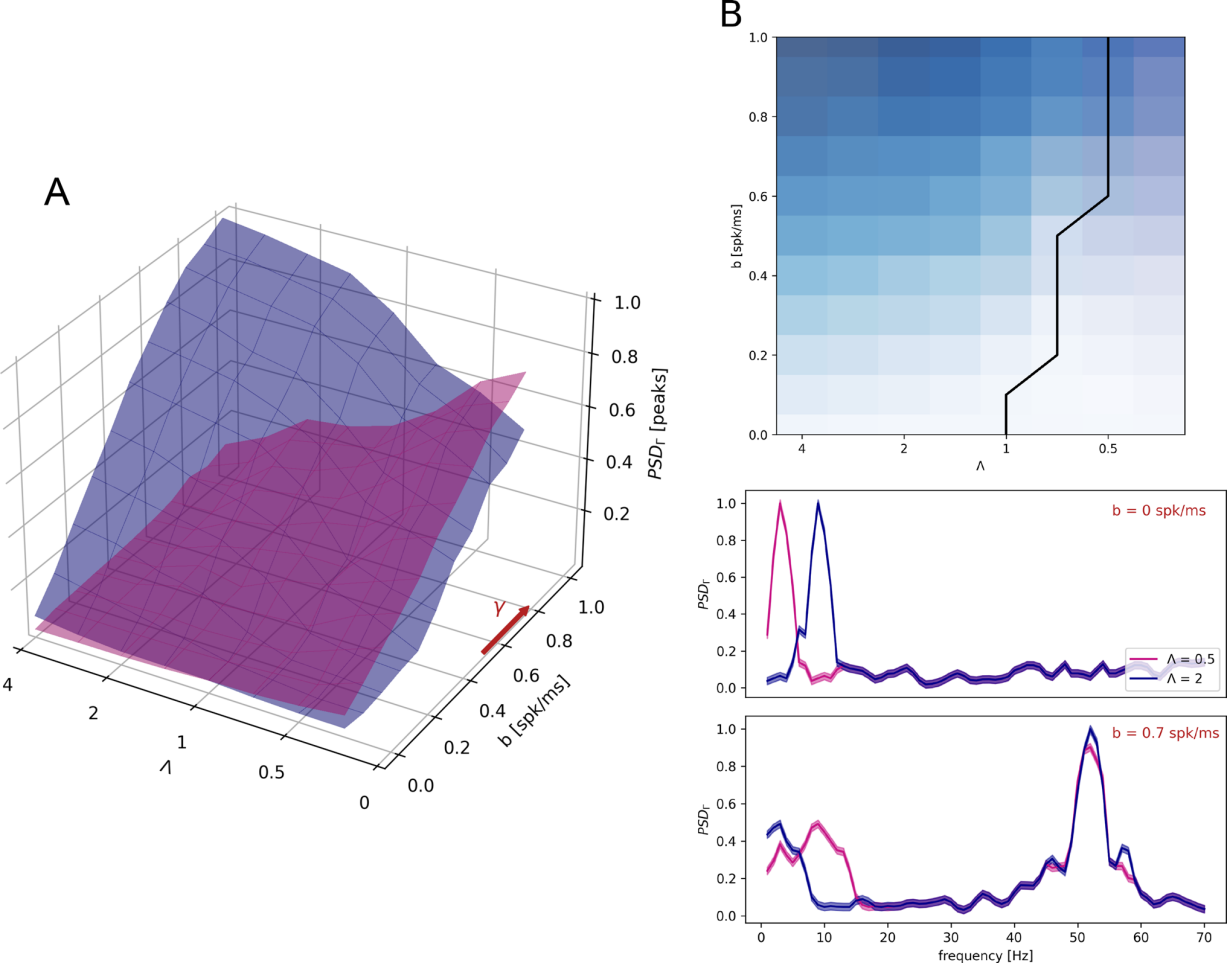
Frequency transfer as a function of network inputs. **A** Cortical power spectral density $\text {PSD}_{\Gamma }$ peaks in the $\delta$ (blue surface) and $\alpha$ (purple surface) when injected with input described in Eq. (11) as a function of baseline *b* and ratio $\Lambda$ between relative amplitudes of the two frequencies of the artificial input. Red arrow represents higher $\gamma$ rhythms increasing in the direction of increasing *b*. The frequency spectrum peaks are in arbitrary units and normalized. **B** Top plot: Projection on the $\Lambda$-*b* plane of intersection between $\delta$ and $\alpha$ surfaces, in blue and purple colormap respectively. The intersection between the surfaces is highlighted in black. Bottom plot: Cortical power spectral density $\text {PSD}_{\Gamma }$ for different values of both *b* and $\Lambda$ as reported on the plots. Results are averaged over 20 different simulations. During all simulations we consider $\nu _{\delta }$ = 3 Hz and $\nu _{\alpha }$ = 9 Hz (Color figure online)

### Modulation of Frequency Response by Internal Cortical Dynamics

The fact that $\alpha$ oscillations are less prominent in the thalamic input to the cortex compared to the whole thalamic LFP (Fig. 4) does not account for the whole extent of the filtering of oscillations in the $\alpha$ range in the cortex. We investigated then if the internal cortical dynamics plays a role in shaping the spectral response properties of the cortex as well. To that end, we consider the dynamics of the cortical network receiving only excitatory inputs from the mimicked ongoing activity of afferent cortical areas (see “Methods”) and from a designed set of inhomogeneous Poisson spike train inputs whose rates $\nu (t)$ were composed by the superimposition of three elements: a baseline constant input, a sinusoidal modulation in the $\delta$ band, and another one in the $\alpha$ band, as follows

11 $$\begin{aligned} \nu (t) = b + a_{\delta }\cos (2\pi \nu _{\delta }t) + a_{\alpha }\cos (2\pi \nu _{\alpha }t) \end{aligned}$$

where *b* is the baseline, $a_{\delta }$ and $a_{\alpha }$ the amplitudes of the two modulations considered, and $\nu _{\delta }$ = 3 Hz and $\nu _{\alpha }$ = 9 Hz their frequencies. To preserve the thalamocortical dynamics studied so far, every cortical neuron receives the same amount of thalamocortical inputs as every thalamocortical connection is substituted by an inhomogeneous Poisson process with the rate given in (11) and the same parameter values for the thalamocortical synaptic current model as listed in Table 2. This artificial input is designed to test the sensitivity of the cortical network to specific spectral contents and allowed us to analyze the cortical response in function of the ratio $\Lambda$ between the amplitudes of the two sinusoidal modulations

12 $$\begin{aligned} \Lambda = \frac{a_{\delta }}{a_{\alpha }} \end{aligned}$$

We studied how the cortical spectral response changes with respect to parameter variations in the 2D space $(b,\Lambda )$ (Fig. 5A). We found that spectral transmission was symmetric only in the lack of external stimulus, while it favoured $\delta$ respect to $\alpha$ components for any non-zero value of the external stimulus. For instance , for *b* = 0 and $\Lambda$ = 0.5 the $\alpha$ component dominates in the cortical activity, while for $b>$ 0 the $\delta$ component is almost as strong as $\alpha$ oscillations, and the opposite does not occur. Overall, the stronger the baseline activity, the higher the peak in the $\gamma$ range and the larger the interval in which the $\delta$ activity dominates in the cortex, even if injected with a smaller amplitude than $\alpha$-activity (Fig. 5B). These results show that filtering of the $\alpha$ spectral components depends on the cortical sensitivity, and is more evident when the input baseline is strong, and hence the cortical spectrum is dominated by $\gamma$ activity.

### Spectral Role of the Relative Weight of Thalamic Inputs to Cortical Populations

**Fig. 6:**
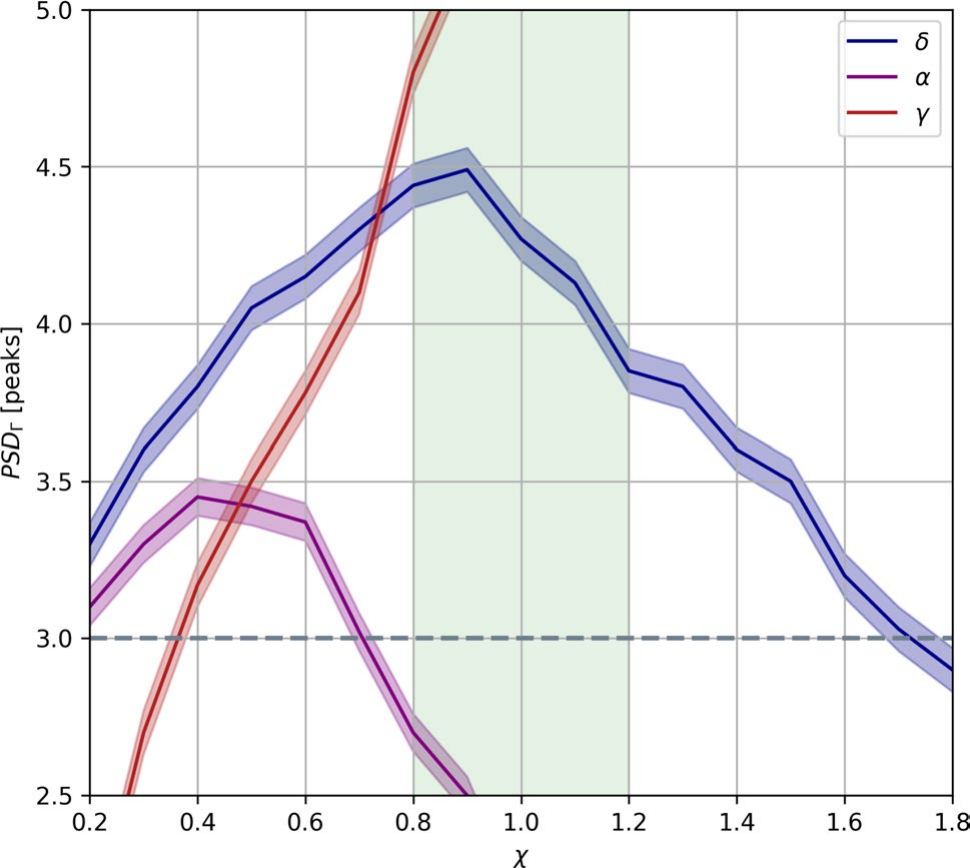
Spectral cortical and thalamocortical architecture. Modulation of the cortical $\text {PSD}_{\Gamma }$ as a function of control parameter $\chi$. Lines indicate power peaks for different frequency bands (see legend). Light green indicates the reference region. Power under the grey dashed line are not associated to peaks. The spectrum peaks are in arbitrary units. Results are averaged over 20 simulations with different noise realisations

It can be observed that when the PSD of the cortical activity contains a high component in the $\gamma$ range, the $\alpha$ component within the thalamic input is substantially, whereas the $\delta$ component is still present. We investigated whether the cortical response depended on ongoing cortical rhythms also during actual thalamic stimulation rather than the simplified external stimulus described in the previous subsection. In particular, we examined the dependence of the cortical spectrum on the relative weight of thalamocortical inputs to cortical populations. First we defined $\chi _0$ as the ratio of the thalamocortical synaptic conductances considered so far in the study

$$\begin{aligned} \chi _0 = \frac{\bar{g}_{py,tc}}{\bar{g}_{int,tc}} \end{aligned}$$

in particular, $\bar{g}_{py,tc}$ = 3.28 nS and $\bar{g}_{int,tc}$ = 4.44 nS (see Table 2). To examine the effect of the relative weights of thalamocortical inputs, we varied the values of the thalamocortical synaptic conductances, and we investigated how this affected the spectral characteristics of cortical LFP signal. To this end, we defined a control parameter $\chi$ as the inverse of the previously defined $\chi _0$ multiplied by the ratio of the new thalamocortical synaptic conductances

13 $$\begin{aligned} \chi = \frac{\bar{g}_{int,tc}}{\bar{g}_{py,tc}}\frac{g_{py,tc}}{g_{int,tc}} = \frac{1}{\chi _0}\frac{g_{py,tc}}{g_{int,tc}} \end{aligned}$$

where we let the parameters $g_{py,tc}$ and $g_{int,tc}$ vary. We computed the peaks of cortical $\text {PSD}_{\Gamma }$ in the $\delta$, $\alpha$ and $\gamma$ bands as a function of the control parameter $\chi$ (Fig. 6A). We found that changes in the input balance have a profound effect on the propagation of the different frequency bands from thalamus to cortex observed so far. When INT neurons receive most of the inputs ($\chi$ < 1), the cortex becomes progressively closer to a faithful reproduction of the thalamic activity (including the $\alpha$ spectral component), up to the disappearance of $\gamma$ component in the spectrum. When PY neurons receive most of the input ($\chi$ > 1) the cortex becomes progressively dominated by the $\gamma$ oscillations, due to the interplay with the INT neurons disrupting lower frequency oscillations (Brunel and Wang 2003). In the balanced condition ($\chi$ $\sim$ 1), it is possible to observe the presence of both the $\gamma$ spectral component and the $\delta$ one, with a clear reduction of the PSD component in the $\alpha$ range (Fig This indicates that a balanced input to the thalamus is the optimal way to selectively block only the non-informative $\alpha$ component generated by the thalamic internal activity (Steriade et al. 1993; Belitski et al. 2008), so that $\gamma$ rhythms are thus the decisive factor of this mechanism.

## Discussion

Our in-silico investigation shows that thalamocortical connectivity modulates spectral transmission between the two areas in a way that optimizes spectral information transmission. We observed that thalamic oscillations in the $\delta$ band [1–4 Hz] and in the $\alpha$ band [8–12 Hz] have remarkably different effects on cortical activity. In fact, the latter embodies slower $\delta$ with respect to thalamic $\alpha$ rhythms while showing another frequency-peak in the higher $\gamma$ band [30–80 Hz]. Under external stimulation, the cortex reproduces the thalamic $\delta$ band and locally generates $\gamma$ rhythms proportionally to the input intensity, while the thalamic spectra show an enhancement in the $\beta$ band [20–30 Hz]. This model reproduces well-known cortical $\gamma$ peaks in response to increasing external inputs (Brunel and Hakim 1999; Brunel 2000), but it also differentiates functional roles of thalamic spectral components in transmitting information to the cortex. In particular, it suggests that thalamic $\delta$ rhythms act as a local *clock* conveying information about internal thalamic states through the activity of the reticular nucleus, in agreement with experimental findings (Lewis et al. 2015). Our results suggest that external stimulation modulates high-frequency activities over a low-frequency background activity, keeping $\delta$ rhythms as a communication channel, similarly to what happens during non-REM sleep (Dang-Vu et al. 2011). On the other hand, thalamic activity in the $\alpha$ band play a minor role in communication to the cortex (Belitski et al. 2008), as they are locally generated in the thalamus. By investigating the mechanisms behind spectral selectivity, we found that both thalamocortical afferents and cortical responsiveness play a role in shaping spectral information transmission, as we dissect in what follows.

Firstly, thalamocortical afferents are composed only by projections from thalamocortical relay (TC) neurons to cortical populations. However, the thalamic LFP is mainly contributed by reticular (RE) projections, which embody stronger $\alpha$ rhythms (Barardi et al. 2016) arising from an inhibition-driven interplay between RE and TC cells, as observed experimentally (Steriade et al. 1993, 1987). Consequently, the spectral composition of thalamocortical inputs reveals that only a fraction of $\alpha$ rhythms is really conveyed to cortical populations. Our results strengthen the hypothesis that spindles, commonly observed in the thalamus (Steriade et al. 1993) are a localized feature of the thalamic network which serve as modulators of information trasmission from sensory input to the cortex through tuning of thalamocortical relay modes (Sherman 2001). Indeed, spindles are locally generated in the thalamus and typically arise from the interplay between TC and RE neurons (Steriade 2005). In particular, RE neurons shape the computational capabilities of the thalamus through the low-threshold bursting behavior of TC neurons. Reticular neurons hyperpolarize TC neurons leading to de-inactivation of T-type calcium channels, what switches TC activity from tonic to bursting (Sherman 2001), leading to spindle oscillations. Many attempts have been made in order to reproduce spindle activity in computational models of thalamic circuitry. One of the first works on TC-RE was developed more than 20 years ago (Destexhe et al. 1993). Recent works describe spike-wave discharges in sleeping thalamus by means of neural field models (Fan et al. 2017). Other late works are based on modeling thalamic cells using single-compartment conductance-based model with several ionic currents. One example is (Li et al. 2017), in which the authors suggest that intrinsic thalamic activities are able to generate oscillations at different timescales, from slow waves to gamma rhythms. However, state-of-the-art modelling of thalamic oscillations lacks simpler yet efficient integrate-and-fire network models. Apart from our work (Barardi et al. 2016) we are only aware of a single previous work regarding Up-Down oscillatory activity in thalamus and cortex (Destexhe 2009).

Secondly, we characterized an intrinsic mechanisms of frequency selection as a function of the cortical dynamical state. Previous works report that cortical $\gamma$ oscillations respond in a resonant manner, implying the proximity of cortical dynamics to a Hopf-bifurcation (Xing et al. 2012; Barbieri et al. 2014). We extend this by showing that the absence/presence of $\gamma$ rhythms shape the responsiveness of cortical network with respect to external modulation in the $\delta$ and the $\alpha$ band. In particular, the presence of strong stimulus-driven $\gamma$ oscillations seems to dampen cortical resonance in the $\alpha$ range while acting as a slow envelope in the $\delta$ range. When $\gamma$ rhythms are absent, the cortical network behaves in the asynchronous irregular (AI) (Brunel 2000) state and thus shows a symmetrical frequency-response portrait.

Finally, we also investigated the role of cortical synaptic parameters in shaping the cortical response, focusing on thalamocortical synaptic conductances. We showed that, in our modeling description, a kind of *democratic* thalamocortical relay to both cortical population through TC neurons activity is optimal for information routing. This input balancing reflects the balanced-network approach used in modeling cortical networks, which is able to reproduce sub- and supra-threshold fluctuations similar to recordings in vivo (Haider et al. 2006; Buzsáki and Wang 2012), and is thus a promising theoretical framework to study cognitive processes in the cortex (Shew et al. 2011; Denève and Machens 2016). In fact, cortical $\gamma$ rhythms arise from such a balance scheme as a collective phenomenon resulting from the interplay between excitation and inhibition (Brunel and Hakim 1999). In our case, balanced inputs correspond to thalamocortical afferents sending stronger inputs to interneurons respect to pyramidal neurons in order to maintain their internal balance. Such scheme is in agreement with recent experimental observations (Sedigh-Sarvestani et al. 2017; Mazade and Alonso 2017). Seminal works by Sherman such as (Murray Sherman and Guillery 1996) highlight that thalamocortical inputs to the cortex are characterized by large and sparse connectivity, able to send powerful information throughout the highly interconnected cortical circuitries. Moreover, thalamocortical relays seem to account for 5–25$\%$ of the excitatory connections within the cortex (Mazade and Alonso 2017). In vivo recordings suggest that thalamocortical afferents seem to target in equal numbers to both the cortical excitatory and inhibitory populations (Sedigh-Sarvestani et al. 2017). To the best of our knowledge, our work is the first effort in modelling the mesoscopic activity arising from such a sparse but symmetric architecture by means of a simple spiking neural network.

Investigations of thalamocortical activity have been done by Bazhenov et al. Bazhenov et al. (2002) with a model based on *in vivo* recordings, which was able to reproduce Up-Down oscillations. However, the authors considered few neurons with complex Hodgkin–Huxley models and fixed connectivity scheme. Later, Izhikevich and Edelman Izhikevich and Edelman (2008) developed a large-scale model of remarkable complexity with several cortical layers, synaptic plasticities and anatomical features. This model is based on the powerful-and-sparse approach for thalamocortical feedforward afferents and on a simple (integrate-and-fire) Izhikevich model for single neuron dynamics (Izhikevich 2003). By using mean-field approaches, important work has been done in understanding the effects of brain stimulation on cortical alpha rhythms (Hutt et al. 2018; Lefebvre et al. 2017) and the interaction between the basal ganglia and the thalamocortical system (van Albada and Robinson 2009). Such mean-field approximations are useful to understand the role of the spectral thalamo-cortical interactions to improve brain stimulation and shed light on the mechanisms of general anesthesia (Hutt 2019). Recently, higher levels of detail have been reached in modeling thalamic and cortical oscillations (Ching et al. 2010) and local field potentials with 3D distribution models (Hagen et al. 2017). Despite of these relevant works, a simple and manageable model able to test novel experimental findings such as (Mazade and Alonso 2017; Sedigh-Sarvestani et al. 2017) is a necessary step to have a shot at describing thalamocortical dynamics while easily investigating different parameter settings.

Computing local field quantities from the activity of spiking network models gives a wider view of the relationship between neuronal interactions and collective phenomena such as oscillations and synchronization (Buzsáki and Draguhn 2004). In this respect, it would be of interest to test experimentally the relationship between cortical balance and spectral content transmission by means of cortical stimulations and LFP recordings. As we focused on particular thalamic input emerging from our thalamic model (Barardi et al. 2016), a possible further development would be to design different artificial inputs and analyze the corresponding cortical response as a function of both stimuli features and the internal dynamical state of the network. We stress that here we have focused on a purely feedforward model of interaction from a local thalamic area to a primary cortical circuitry of the related sensory system to investigate feedforward transmission of oscillations (as in Brown et al. (2020)). Therefore, one relevant limitation of the model discussed in the present work is the inability to generate proper intermittent sleep spindle oscillations. This is due to the fact that we adopted a purely feedforward thalamocortical model with no corticothalamic feedback, which is necessary to generate sleep spindle oscillations. This work focuses on thalamocortical spectral transmission, and we aimed at assessing the way the different frequency bands are filtered independently from the way they are generated. However, in follow-up studies we will include feedbacks, as these are likely to reinforce transmission in the alpha range and compensate to some extent the mechanisms highlighted in in the present work.
